# Group cognitive behavioural therapy for women with depression: pilot and feasibility study for a randomised controlled trial using mixed methods

**DOI:** 10.1186/1471-244X-11-82

**Published:** 2011-05-13

**Authors:** Helen Cramer, Chris Salisbury, Joel Conrad, James Eldred, Ricardo Araya

**Affiliations:** 1 Academic Unit of Primary Health Care, School of Social and Community Medicine, University of Bristol, Canynge Hall, 39 Whatley Road, Bristol, BS8 2PS, UK; 2 Avon and Wiltshire Mental Health Partnership NHS Trust (AWP) Redgables Hilperton Road Trowbridge, BA14 7JE, UK; 3 Avon and Wiltshire Mental Health Partnership NHS Trust (AWP) Callington Road Hospital Bristol BS4 5BJ, UK; 4 Academic Unit of Psychiatry, School of Social and Community Medicine, University of Bristol, Oakfield House, Oakfield Grove, Clifton, Bristol BS8 2BN, UK

## Abstract

**Background:**

Group Cognitive Behavioural Therapy (CBT) may provide a means of improving mental health among people with depression but few studies have explored its effectiveness. Our aim was to examine the feasibility and acceptability of a randomised controlled trial of a group intervention based on CBT principles for women with depression in primary care.

**Methods:**

Women aged 30 to 55 years were recruited and randomly assigned to either 12 weeks of the group intervention or usual care (control). The group intervention was based on a manual and used CBT and problem solving principles with weekly topics including raising activity levels, spotting and catching negative thoughts, problem solving and relaxation. Women were recruited from deprived areas of Bristol. The groups were run by facilitators with some experience and background in group work and one weeks training in use of the course manual. Assessments of mental health were made using measures including the PHQ-9. Follow-up was at 3 and 6 months after the intervention. Qualitative methods were used to support the design of the intervention and to help understand issues of acceptability and feasibility. Interviews were conducted with all participants at baseline and at 3 and 6 months although detailed qualitative analysis was based on a purposive sample of 20 participants at the 3 time points.

**Results:**

Of the 86 participants assessed for eligibility, 52 were allocated to the intervention arm and 21 to the control group. The intervention was delivered according to the manual despite the limited training of the facilitators. The intervention was received favourably by participants and facilitators, with good attendance at sessions for those who engaged with the intervention. Follow up rates at 3 and 6 months for women in both the intervention and control arms were also good. The trial methodology used was appropriate and feasible.

**Conclusions:**

This study showed that a randomised controlled trial of group CBT for women with depression is feasible and the intervention is acceptable, and may possibly prove to be effective in a larger trial. The cost effectiveness of group CBT for depression should be explored further in a full trial.

**Trial registration:**

NCT00663078

## Background

Depression and anxiety disorders are common in primary care, especially among women [1,2]. General practitioners (GPs) are encouraged to recognise patients with depression but they often find themselves with little to offer to patients with moderate or minor depression. NICE guidelines recommend that depressed patients should be treated with brief individual psychological interventions as first line treatment, with group therapies being an option for people who prefer this [3]. Since the availability of therapists is limited, group-based approaches may offer advantages over individual therapy. Group based CBT therapies may potentially be more cost effective than individual CBT [4,5], reaching more people and having additional advantages such as providing mutual support and encouraging good imitative behaviour [6]. However, the evidence base for group CBT is much less strong than it is for individual CBT [7].

This paper is based on a feasibility and pilot study of a group-based intervention in primary care for women with depression living in socially deprived areas in the United Kingdom. The aims of this paper are to describe the development of the intervention and methods of data collection, ascertain the feasibility of recruitment, assess the feasibility and acceptability of the group intervention and to make a preliminary assessment of its effectiveness. This study encompasses Phases I and II of the MRC framework for evaluation of complex interventions which are necessary preliminary steps towards conducting a full trial of group CBT [8].

## Methods

This study used both qualitative and quantitative methods to optimise the intervention, test feasibility and pilot the randomised controlled trial. The target population was women from disadvantaged areas, where the prevalence of depression is often highest [9]. This trial was designed exclusively for women, as female only groups have shown better recruitment and lower dropout rates [10,11] and, it was thought that mutual support might be achieved more effectively in a single sex group with a restricted age range. The recruitment focused on two disadvantaged areas of Bristol: an inner city area with large Black and minority ethnic populations and a more peripheral urban area with predominantly white residents.

Ethical approval was given by Frenchay Research Ethics Committee (07/H0107/60).

### Recruitment

Study inclusion criteria were: women aged 30 to 55 with clinical depression according to the Patient Health Questionnaire (PHQ-9). Exclusion criteria were: severe depression (this criterion was later relaxed in the second round of groups); drug or alcohol abuse; currently attending specialist psychiatric services (including psychotherapy); or being unable to speak English. Current or past use of antidepressants or benzodiazepines was not an exclusion criterion.

Local GPs, mental health professionals and community organisations such as health training teams and domestic violence organisations were all invited to refer potential participants. Posters and fliers were distributed around community venues to encourage women to refer themselves. The information circulated did not mention the term 'depression', but asked women to get in touch if they felt 'stressed' or 'not able to cope'. Medical notes searches were also conducted in 5 GP practices to identify potential participants who had a diagnosis of depression or anxiety, were currently taking antidepressants, or with a recent PHQ-9 score between 5 and 24. Practices wrote to women identified asking them to contact the research team if interested in participating. The 5 practices involved had 30,383 registered patients, with individual practice list sizes ranging from 4,848 to 7,300.

Intensive recruitment was conducted over a restricted period of two months in each area to ensure that potential participants were not left waiting too long between being recruited and the groups starting. Comparison of the feasibility and efficiency of each of the recruitment methods was done by maintaining a record of all referral sources for all potentially interested people contacting the research office. Information about recruitment was also collected during follow up calls to recruiters and a limited number of interviews.

There was a three stage process of recruitment. After an expression of initial interest by a potential participant through one of the routes detailed above, further information about the study was given over the telephone. For those still interested after the telephone conversation, detailed written information and consent forms were sent and the person invited to attend an appointment with the researcher. At this face-to-face assessment, potential participants completed a PHQ-9. Those with a score of between 10 and 20 (first round of groups) or 10 and 24 (second round) and no reason for exclusion were considered eligible for the study. PHQ-9 scores of 5, 10, 15, and 20 represent mild, moderate, moderately severe, and severe depression, respectively [12]. We increased the upper threshold for the PHQ-9 in the second round of groups since experience from the first recruitment round suggested that the lower threshold excluded some women who appeared suitable for the group intervention. All eligible participants were then asked to provide written consent, complete a baseline questionnaire, and to participate in a semi-structured qualitative interview. Details of the measures included in the baseline and follow-up questionnaires were as follows:

• Patient Health Questionnaire [13]

• Anxiety symptoms: Beck Anxiety Inventory [14]

• Health Status: Physical and Mental component scores from the Short Form-12 Questionnaire [15]

• Dysfunctional thoughts: shortened Automatic Thoughts Questionnaire [16]

• Social support: shortened Medical Outcome Study Social Support Survey [17]

• Recent health service use

• Socio-demographic characteristics e.g. ethnic group, household income, housing tenure.

After baseline assessment, participants were randomised to the intervention or control arm.

### Randomisation

Concealed allocation of eligible participants to either the intervention or usual care arm was achieved using an automated randomisation system accessed via the web. Minimisation was used to ensure balance in PHQ-9 score (≤15 vs. ≥ 16) and GP practice. The allocation ratio was 2:1 in favour of the intervention arm in order to ensure that groups were viable. Participants were randomised and informed of their treatment allocation at the end of baseline interview. The allocation was not concealed from either the participants or the researcher, although analysis was conducted blind to allocation.

### Intervention

Building on the findings from previous studies [10,11,18,19] the intervention was based on principles derived from CBT and problem-solving approaches. Using homework and skill based approaches the group intervention sought to empower participants through improving their self-management skills. It is important to note that the course was designed to be used by non-professional facilitators following a manual. It should be considered as an attempt to design a low-cost group intervention based on CBT principles which could be made widely available rather than being equivalent to individual CBT from a trained therapist.

*Facilitators and groups: *A pair of trained facilitators delivered the intervention. There was a deliberate strategy to recruit facilitators who were not professional therapists in order to make the group intervention widely available, to ensure that facilitators were socially connected to the target population in deprived areas, and to keep the cost of the intervention low and therefore to maximise its potential cost-effectiveness. Facilitators were recruited via advertisement in local newspapers, community magazines and word-of-mouth. There were over 150 applicants. Ten (female) applicants were recruited. Prior to the study none of the facilitators had received CBT training but most had counselling skills, some had experience of group work and one had lived experience of mental ill health. Facilitators received a 5-day training course from experienced CBT trainers (JC and RA). Facilitators also continued to receive regular fortnightly supervision from the CBT trainer (JC). Four intervention groups were run in 2 rounds in 2 locations, with 1 morning and 1 evening group in each location.

*Session content: *The intervention consisted of 12 sessions delivered over 10 consecutive weeks with 2 booster sessions after a gap of 2 to 4 weeks at the end. The course content was based on previous courses and interventions [18,20-22] and is shown in summary below:

1. Introduction and group rules

2. Checking activity levels

3. Raising activity levels

4. Catching negative thoughts

5. Balancing negative thoughts

6. Managing anxiety

7. Relaxation

8. New Ways of Solving Problems

9. Integrating and catch up session

10. Making a personal plan

11. Booster session I

12. Booster session II

All participants received a free manual which described the content of each session, along with exercises and space for notes. Considerable attention was given to ensuring the course manuals were clear, attractively laid-out and in a language acceptable to targeted users. Facilitators followed a similar course manual with instructions and suggested timings. Fidelity of the intervention was ensured by regular supervision and observation of one session for each of the groups. Two members of the research team (JC and HC) assessed the delivery and quality of the intervention by using a modified version of the CBT rating scale (CTSR), adapted to make it suitable for a group [23]. Where consensus was not reached a third member of the team (CS) decided.

### Usual care

Participants in the control arm were given an information booklet. This contained details of local support organisations such as local mental health organisations, counselling services, carers groups and Black and minority ethnic services. An information booklet was given in addition to usual care because it was thought that it might improve study retention in the control arm. Participants in both arms were allowed to continue taking (or start) any antidepressant or other medication prescribed by their GP.

### Data collection and outcome measures

Patients were assessed at baseline, and 3 and 6 months after starting the groups. The primary outcome measure was the PHQ-9. Secondary outcomes are listed above. All measures were used at baseline and after 3 and 6 months. Follow-up data were collected either during an appointment, by postal questionnaire or over the telephone.

### Qualitative methods

Qualitative methods were used to support the design of the intervention and to help understand issues of acceptability and feasibility. Interviews were conducted with all (75) participants at baseline and at 3 and 6 months exploring motivation to join the study, own definitions of improvement and experiences of the groups. Subsequent sampling and detailed analysis was done with 20 participants from interviews at baseline, 3 and 6 months (14 in intervention and 6 in control arm at each of the 3 time points). Purposeful random sampling was done to ensure those selected represented a range of illness severity (based on PHQ-9 scores) and high, low or no attendance at groups. In addition, interviews with 2 GPs and 5 community staff were conducted and feedback collected from the facilitators during 2 focus groups and on a weekly basis. Interviews, focus groups and observations were all digitally recorded with consent and transcribed. The interviews were analysed thematically by the constant comparison method and by taking a Framework approach [24]. Some data, such as interviews with recruiters, was organised in Atlas ti, data from participant interviews was organised using Excel spreadsheets and the Framework approach. HC led the analysis, although the credibility of the Framework categories was checked by other members of the team.

### Statistical analysis

We used descriptive statistics such as means, standard deviations and proportions to describe the characteristics of patients allocated to the intervention and control arms at baseline. Three individuals who had been randomised to the control arm were invited to the intervention arm in order to bolster the numbers required to form a viable therapeutic group. These individuals were chosen at random and all agreed to this change. In the analysis these individuals were treated as members of the intervention arm (treatment allocated) because they received the intervention but we also performed sensitivity analyses in which we analysed these individuals according to their randomisation to account for this protocol alteration. Because this was a pilot study, not expected to provide definitive evidence about effectiveness, no power or sample size calculation was performed. Comparisons across groups were done using linear and logistic regression. The primary outcome variable (PHQ-9) was used as a continuous variable and also as a binary variable (proportion of patients 'improved' (50% score reduction) and 'recovered' (score <10)) to compare groups as allocated to treatment, with adjustment for PHQ-9 baseline scores. Similar regression models were used for secondary outcomes. All these analyses were repeated for primary and secondary outcome variables at 3 and 6 month follow-up periods. Secondary analyses compared participants according to the treatment actually received (on the basis of records of therapy sessions attended). These analyses of complier-average causal effect used instrumental variables in linear regression models for PHQ-9 at each follow-up as a continuous score. We also dichotomised attendance according to whether or not participants attended at least half (six) of the sessions. All statistical analysis was performed using STATA version 9.0.

## Results

### Recruitment

Based on the searches of medical notes, an invitation to participate was mailed to 449 potentially eligible participants (over the 2 month-long recruitment periods) and 123 women responded expressing an interest in the study (27%). Recruitment by medical notes searching was the most successful of the three methods used. When participants were interviewed about recruitment quite a few women who had been sent a study letter following the medical notes search felt that their GPs personally referred them. A few other participants selected by this method were more wary and specifically asked why they had been sent a letter. Most of the GP practices approached agreed to conduct a medical notes search but only two practices volunteered to additionally recruit by patient referral. Few referrals were made by GPs other than via the notes search (7) or by statutory mental health assessment teams (6).

Community referral and self referral were also good sources of recruitment. Thirty potential participants came after seeing a poster, leaflet or talking to someone in a community organisation. Another seven had been directly referred from community organisations. The attitudes of community staff to the study seemed to vary considerably and these attitudes directly related to the level of staff or self-referrals linked to these organisations. For example, representatives of two community organisations referred several women and said in interviews that they welcomed the opportunity to offer their clients an extra service. However, some representatives of other (low-referring) community organisations said that they did not agree with the principle of randomisation, believing that it was unethical because not all the people who expressed an interest in the study would receive the new service. The short period of intensive recruitment also meant that community organisations had little time to plan or invite people to join the groups.

**Figure 1:**
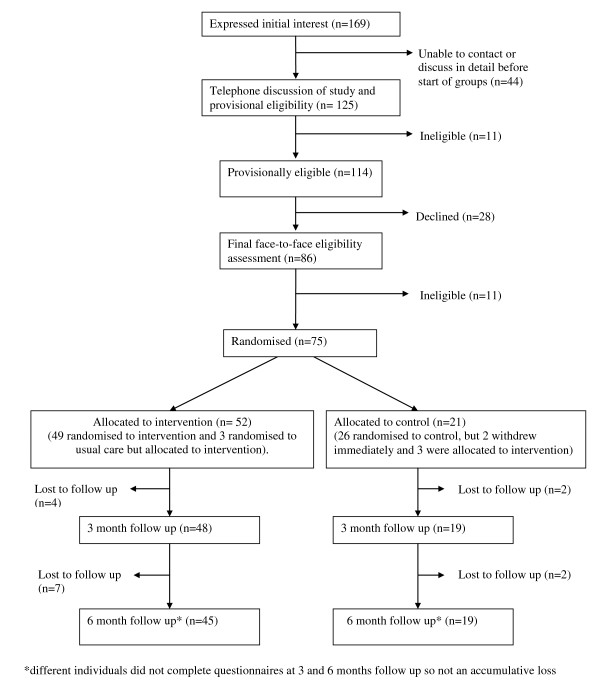
The CONSORT diagram

The CONSORT diagram (see Figure 1) shows the total numbers of participants recruited into the study. The diagram shows initial contact with 169 women of whom 86 were later assessed. Of the 75 eligible participants 49 were randomised to receive the intervention and 26 to usual care. As described above, three women who were randomised to the control arm were re-allocated to the intervention arm to make the groups viable. Two participants allocated to usual care withdrew immediately after being randomised because they had hoped to receive the group intervention. Therefore, 52 women were allocated to the intervention and 21 to usual care.

Some of the losses (44) in potential recruits at the first stage were due to delays in GP practices sending letters out after the notes searches, the short time window of intensive recruitment and that fact that the start date of the groups was immovable. Lack of adequate administrative and recruitment support at crucial times also slowed the recruitment process so that for a significant proportion of women, there was not enough time to contact them and arrange an appointment. As common with other depression studies, a number of women failed to attend their assessment appointments and sometimes it was not possible to re-contact the person and arrange another appointment in the time period available. A small proportion of potential participants were ineligible (11) and through the initial telephone conversation it was established that they were either the wrong age, already receiving psychiatric care, had an addiction problem or lived outside the area of the study. Other reasons for the loss of potential participants and also established during the initial telephone call and grouped under the heading 'declined' included (28 in total): people who could not make the particular dates and times set for the groups; people who realised that they would not be able to attend any groups because of family commitments such as lack of regular childcare; people who decided that on hearing further details they did not want to continue and reasons to the effect that it 'didn't sound right for them'; people who said they did not want to meet other people; and people who only wanted to join if a friend could come too.

When asked in interviews why they were attracted to take part in the study, most women (59/73, 81%) said they were attracted to the study because they needed help to change. Women variously expressed this as wanting to 'move on', 'have a challenge', become 'motivated', get 'out of a rut', or 'be more positive'. The social attractions of the group were mentioned fairly frequently by women but not usually as their primary motivation to joining the study. Women often spoke of feeling isolated and wanting more support and adult company. Women wanted reassurance, to feel that they were not on their own and to meet others who felt the same. The following quotes describe some of the social aspects of groups that were attractive:

I saw the poster, because I do suffer from stress and I would like to find ways of coping with it and I quite fancied being in a women's group actually, I thought perhaps I'd benefit from the support (P47, control arm.)

I do get stressed ...sounded like an opportunity to meet other people and discuss ways of dealing with stress, dealing with negative thoughts...like things that I would say I had going on in my head, quite a lot of the time (P67, control arm).

Because I suffer from depression really bad at times...to see if anybody suffers like I do basically or am I just the 'only one' (P86, intervention arm).

Other motivating factors that were mentioned by several women included a preference for help that did not involve medication and having an alternative option to counselling. Some women expressed altruistic motives to join a research study.

### Baseline characteristics

Baseline characteristics are presented in Table 1 to compare the two arms as they were allocated to treatment. Most participants were white, middle-aged with at least one child at home. Few women were recruited to the study from Black and minority ethnic (BME) communities. In line with the strategy of recruiting women from disadvantaged areas, the mean age of leaving full time education suggests a group with low education and average household income of approximately £14.000 [25]. Most participants were living in rented accommodation and two-thirds were receiving state benefits. Approximately one-third were divorced or separated. There were no major imbalances between arms, in spite of the small sample size. The only noticeable imbalance was for occupational status, with the intervention arm including more employed women. The arms were also very similar in all the clinical variables with only a marginally increased severity on PHQ-9 scores in the intervention arm. The baseline measures suggest that most of the women had moderate to moderately severe depression, as well as moderately high levels of anxiety. As expected, given the small number of women reallocated, the results when comparing arms as originally randomised were very similar to those when comparing arms as finally allocated.

**Table 1:** Characteristics of the sample according to allocation to treatment: balance between arms

BASELINE VALUES	N	Intervention	N	Control
Age: mean (SD)	52	42.71 (6.67)	21	41.86 (6.70)

Number of children at home: mean (SD)	52	1.02 (0.94)	21	0.95 (0.97)

Age of leaving full time education mean (SD)	49	17.71 (5.20)	20	17.60 (2.52)

Annual Income: mean (SD)	50	15,841 (11,925)	19	14,299 (8,417)

Marital status				

Single	22	42%	7	33%

Co-habiting	10	19%	4	19%

Married	6	12%	2	10%

Divorced or separated	14	27%	8	38%

Ethnic group				

White/White British	46	89%	18	90%

Other ethnic group	6	11%	2	10%

Accommodation				

Owner-occupied	16	31%	10	48%

Rented	35	69%	11	52%

Employment				

Employed	23	44%	6	29%

Unemployed	4	8%	0	0%

Long term sick	13	25%	7	33%

Looking after family/home	12	23%	8	38%

Receive any state benefits				

Yes	31	65%	13	65%

No	17	35%	7	35%

**Clinical Variables**				

PHQ-9 mean (SD)	52	13.6 (3.1)	21	12.7 (1.9)

Anxiety mean (SD)	52	24.6 (12.1)	21	23.9 (11.4)

Automatic thoughts mean (SD)	49	24.7 (7.7)	21	24.1 (5.9)

Social support mean (SD)	49	21.3 (8.7)	20	22.9 (10.0)

SF-12 Physical component mean (SD)	51	47.9 (10.7)	21	48.8 (13.6)

SF-12 Mental component mean (SD)	51	29.5 (9.8)	21	26.2 (7.8)

Proportion taking antidepressants	52	38.5%	20	35%

#### Primary outcome measures

This analysis was done using the data according to allocation to treatment. As seen in Table 2, PHQ-9 mean scores were lower for the intervention arm compared with the control arm at 3 and 6 months follow-up, although this difference decreased over time and none of the differences reached a significance level of p < 0.05. Similarly there was a trend in favour of the intervention arm when the scores were analysed as a binary variable in terms of improvement (a decrease of at least 50% from the baseline score) or recovery (values below 10). These differences in proportions were large at three months follow-up but with wide confidence intervals given the small sample sizes. At six months all these differences became markedly attenuated. The attenuation of differences in improvement seemed to be explained mostly by a large improvement in the control arm (10% to 38%). The proportion recovered was larger than those improved, something that may be explained by the low starting values (see Table 1).

**Table 2:** Primary outcome results: Comparison across arms (observed data only)

	3 months					6 months				
	**Intervention**		**Control**		Regression*	**Intervention**		**Control**		Regression*

	**N**	**Mean (sd)**	**N**	**Mean (sd)**	**B coefficient (95% CI) P value**	**N**	**Mean (sd)**	**N**	**Mean (sd)**	**B coefficient (95% CI) P value**

PHQ-9 continuous	48	10.0 (6.2)	19	11.5 (5.4)	-1.57 (-4.87 to 1.74) p 0.35	45	10.0 (7.7)	19	11.2 (6.6)	-1.18 (-5.32 to 2.97) p 0.57

	**N**	**%**	**N**	**%**	**Odds Ratio (95% CI) P value**	**N**	**%**	**N**	**%**	**Odds Ratio (95% CI) P value**

PHQ-9 Recovered (Binary <10)	48	58%	19	32%	3.12 (1.00 to 9.72) p 0.05	45	51%	19	42%	1.56 (0.51 to 4.71) p 0.43

PHQ-9 Improved (<50% from baseline)	48	35%	19	10%	4.21 (0.83 to 21.23) P 0.08	45	47%	19	38%	1.21 (0.69 to 2.11) P 0.51

* All regression analyses were adjusted for baseline scores

In order to test more accurately the impact of the intervention we estimated the effect of the number of sessions attended on the main outcome (PHQ-9 score) as a continuous and binary variable. Table 3 presents the results from the complier-average causal effect analyses for the continuous PHQ-9 at 3 and 6 months. These results show that there were small reductions, especially at 3 months, in PHQ-9 scores according to the number of sessions attended. These reductions were more noticeable when using a pre-defined threshold of a minimum of 6 sessions attended, with a drop of 2.47 points among those who completed at least 6 sessions compared to those who attended fewer sessions. Nonetheless it is important to emphasise that none of these estimates was significant at a p value of <0.05.

**Table 3:** Instrumental variables analyses of primary outcome (PHQ-9) at 3 and 6 months (observed data only)

	PHQ-9 score at 3 months	PHQ-9 score at 6 months
Total number of sessions	-0.24 (-0.73 to 0.26), p = 0.34	-0.18 (-0.82 to 0.45), p = 0.56

Optimum number of sessions(>5)	-2.47 (-7.51 to 2.57), p = 0.33	-1.88 (-8.34 to 4.58), p = 0.56

* All regression analyses were adjusted for baseline scores

#### Secondary outcome measures

Anxiety scores were almost equal in both arms and at both time points (see Table 4). Physical functioning (SF12 physical component) was similar across arms at 3 months, but better in the intervention arm than the control arm at 6 months. Mental health assessed using SF12 mental component score showed similar results to those obtained with the PHQ9, that is, an improvement at 3 months in the intervention arm but with subsequent attenuation at 6 months. Scores on the negative automatic thoughts questionnaire improved in both arms over time with no meaningful difference between the arms. Similarly, social support scores were almost identical in both arms and across time points.

**Table 4:** Secondary outcome measures: Comparing means across arms (observed data only)

	3 months					6 months				
	**Intervention**		**Control**			**Intervention**		**Control**		

	N	Mean (sd)	N	Mean (sd)	B coefficient† (95% CI) p value	N	Mean (sd)	N	Mean (sd)	B coefficient† (95% CI) p value

Anxiety** (Beck Anxiety Inventory)	46	19.0 (10.4)	19	19.5 (11.1)	-0.22 (-5.97 to 5.52) p 0.94	42	16.1 (10.6)	19	16.9 (13.4)	1.29 (-4.95 to 7.54) p 0.68

Physical component of SF12 score*	45	46.7 (11.8)	19	45.6 (11.0)	1.59 (-3.18 to 6.37) p 0.51	40	49.3 (10.1)	18	41.5 (11.0)	8.05 ( 3.53 to 12.60) p 0.001

Mental component of SF12 score*	45	37.2 (12.2)	19	29.8 (9.0)	6.07 (-0.04 to 12.2) p 0.051	40	36.22 (13.09)	18	32.8 (13.6)	2.15 (-5.13 to 9.44) p 0.56

Process or intermediate measures										

Automatic thoughts ** (Shortened Automatic Thoughts Questionnaire)	45	19.6 (8.7)	19	22.1 (8.0)	-2.88 (-7.29 to 1.54) (p 0.20)	40	18.3 (8.1)	19	18.3 (7.23)	0.42 (-3.75 to 4.59) P 0.84

Social Support* (shortened Medical Outcome Study Social Support Survey)	46	23.8 (9.5)	19	23.1 (9.6)	2.40 (-1.65 to 6.45) (p 0.24)	40	24.1 (10.6)	19	24.1 (9.91)	0.74 (-3.94 to 5.43) p 0.75

^†^All regression analyses were adjusted for baseline scores

* High scores are good

** Low scores are good

### Process measures: Group attendance, fidelity, medication and use of services

In general, attendance to the groups was reasonably good but booster sessions were poorly attended. However, 12 of the 52 participants in the active arm did not attend any sessions at all. The mean number of sessions attended in the intervention arm was 6 (sd 4) but excluding those who did not attend any sessions the mean number of sessions attended increased to 8 (sd 3). At interview, the reasons given for non-attendance by those who did not attend any sessions were related to family role and responsibilities (e.g. unreliable childcare, family crisis and participants feeling too self conscious or ambivalent about the usefulness of the groups).

We decided a priori that a minimum of six sessions were likely to be needed to achieve clinical improvement. Of all women allocated to groups, 31/52 (60%) achieved this number of sessions, while of those who attended at least one session, 31/40 (78%) attended 6 or more sessions. All facilitator pairs were considered to have adhered to the manual ('mostly' or 'fully') when delivering the intervention and all scored above the threshold for competence in pacing, feedback, participant engagement and therapeutic relationship.

At the start of the study 9/52 (17%) of women in the intervention arm and 5/20 (25%) of those in the control arm reported that they were receiving or had recently received counselling. At the end of the first 3 months of the study 16% of those in the intervention arm compared with 37% of those in the control arm reported having received counselling over that period (p = 0.07). The proportion who reported receiving counselling between 3 and 6 months were 13% in the intervention against 44% in the control arm (p = 0.01). The mean number of all health-related consultations over the last 3 months at baseline were 4.8 (4.5) in the intervention arm and 4.2 (4.5) in the control arm, 5.1 (.2) and 4.6 (4.7) at 3 months (p = 0.75) and 3.9 (3.9) and 6.0 (4.3) at 6 months (p = 0.06) respectively.

#### Participant feedback at qualitative interviews

Feedback about the manual was positive and participants reported that they understood most of the material delivered by the facilitators. There was some resistance to homework, a key feature of the CBT approach. Feedback about the group experience overall was overwhelmingly positive. There were numerous examples of how the groups had acted as a catalyst for change including participants taking up new jobs, returning to previous paid or volunteer work, resuming driving and swimming lessons, starting courses and booking holidays. Aspects of the course singled out for most praise included the idea that negative thoughts could be changed, a reminder (and permission) to do more enjoyable things, and the sharing and supportive relationships within the groups. Negative comments about the groups were that the course was irrelevant to their lives, that change was not possible, that listening to others' problems had made them feel worse and that they felt even more alone once the course had finished.

## Discussion

### Summary of main findings

This study demonstrated that a full scale randomised controlled trial to examine the effectiveness of a group intervention based on CBT principles for women recruited from primary care would be feasible and acceptable, and such a trial is now needed. Although confidence intervals were wide due to the small sample size, the improvement observed in the primary clinical outcome suggests that the group intervention may be more effective than usual care in the short term but maintaining gains in the longer term remains a challenge. Interestingly there was a noticeably higher proportion of women receiving counselling amongst those in the control group, suggesting that offering groups may reduce the need for individual counselling.

It is notable that a greater proportion of women in the intervention arm recovered from depression at 3 months follow-up, compared with the control arm, yet the difference in improvement in mean score was fairly small (-1.57, equivalent to an effect size of 0.27). This may be because many women had scores only just above the threshold for depression at baseline and a slight reduction of score took them over the threshold into recovery. Despite the statistical limitations of using a dichotomous approach to analysing the PHQ9, the concept of recovery is probably more meaningful to both women and their doctors than one of a difference in PHQ9 score.

This study highlighted that searching GP records seemed the best method of recruitment. Randomising between a psychological intervention and usual care is achievable but it is essential to ensure that women fully appreciate that randomization to the control arm is a possibility in order to reduce subsequent drop-out from the control arm. The course and manual were acceptable and appreciated by participants and facilitators delivered it faithfully and as intended. Group attendance was adequate except for the booster sessions. However a substantial minority of women did not attend any group sessions, suggesting that people make up their minds about participation rapidly.

### Strengths and limitations of the study

This was a pilot study designed to assess the feasibility of a trial and to optimise the intervention. It provides an initial indication that the intervention may be effective, but the small sample size means that it lacks power to detect clinically meaningful differences. It is important to conduct a fully powered trial, and this pilot study provides useful data to inform sample size calculations for this. The study was also limited because we did not include a formal diagnostic interview to compare with the brief PHQ-9 assessment tool. However, the PHQ9 is widely used within UK primary care as a basis for diagnosis and treatment of depression, so our approach has good external validity. We collected some data about health service resource use, but we did not collect enough data to conduct an economic analysis. The strengths of the study lie in its successful recruitment strategy with wide eligibility criteria without reliance on referral from health professionals. The intervention concept itself was simple and attractive, within a context of few alternative free services. The use of lay facilitators following brief training and with limited supervision appeared to be successful. It was also encouraging to see a large number of people applying for these posts, suggesting that it would be possible to scale up this intervention without difficulty. The anticipated problems of low interest and recruitment in group interventions, high dropout rates and loss to follow up were all overcome. The manual was accessible to participants and its model of delivery workable with a high level of overall satisfaction among users and good fidelity among therapists. The use of qualitative data collection within the pilot trial design was fundamental to an understanding of the intervention's feasibility and acceptability, and providing ideas about ways to improve both the intervention and conduct of the trial.

### Feasibility of recruitment

Recruitment into trials for mental health interventions is notoriously difficult [26]. Gaining interest in this study was not problematic; the main challenge was to assess and recruit enough people rapidly enough over a short period in order to form groups with sufficient numbers. Although one of the early groups nearly floundered from low initial numbers, the overall success and relative ease of recruitment seems to support the recruitment strategies chosen. It appeared that the simplicity of the intervention concept (attending a group with other stressed women and being taught skills to cope better) helped participants and recruiters to understand and promote the groups. The wide and inclusive criteria for the study helped to secure a large pool of potential participants. In addition, wide PHQ-9 threshold scores (between 10 and 20/24) meant that relatively few participants were subsequently excluded at the eligibility screening stage. Few women were recruited from BME groups, suggesting that additional recruitment strategies may be necessary for this group, probably involving building stronger initial links with BME organisations. Although many women were interested in attending a 'talking therapy' group, only a small number said that they were interested in CBT specifically. Many women said that they wanted alternatives to medication or felt attracted by the idea of meeting other women experiencing similar issues. There seemed to be good support among users for research on depression, especially if it did not involve medication. Finally, the relative ease of recruitment to the study seems to be supported by having used a range of methods, especially the medical notes searches and not being reliant on referral from GPs or other agencies.

### Feasibility, quality and acceptability of CBT delivered in groups

For a large scale trial it is important for an intervention to be reproducible. The manual developed was well-received by participants and provided a strong basis for standardizing the intervention allowing its delivery by facilitators with minimal training and support. Although we designed this course so that it could be led by facilitators with little experience of delivering psychological therapy, a large group of skilled facilitators with experience in counselling and/or leading groups in other contexts applied for the jobs. As the intervention was delivered by people without specialist CBT training there may be concerns raised as to the quality of the therapy being offered. From the observed sessions the team concluded that facilitators delivered the course contents as instructed, even though their skills were not comparable with fully trained CBT therapists.

Attendance at the groups was variable, but it was reasonably good amongst those who attended at least one session. In keeping with findings from other studies, dropout rates were marked between first and second sessions when individuals make up their minds about their interest in this kind of intervention. With hindsight perhaps a bigger effort to explain in greater detail the type of treatment might have helped to reduce initial dropouts as well as providing additional individual attention. Improvement in retention to the poorly attended booster sessions could be addressed by the final sessions being brought closer, and not happening after a break of four weeks. Attending more sessions tended to be associated with an improved primary outcome. The qualitative research indicated that the level of attendance achieved appeared to be largely reliant on the support and encouragement of the facilitators and other group members. Participants' experiences in the groups seemed to be generally good and there was some evidence of relationships and support lasting after the groups had finished.

### Understanding mechanisms of change

No changes were observed in what we had construed as intermediate process variables. Neither responses to the social support questionnaire nor the dysfunctional thoughts questionnaire showed differences between arms. Interestingly these findings run contrary to the qualitative findings which suggested that social support for most participants had increased during the course of the intervention, especially from other members in the group. Likewise, participants reported significant improvements in their negative and balanced thinking in the qualitative findings. Although the sample size is small, these discrepancies may indicate the need to re-evaluate the appropriateness of these measures in a larger study.

### Comparison with previous studies: clinical effectiveness

A small number of previous studies have assessed the effectiveness of similar group based psychological therapies. For example, the European ODIN study compared 6 sessions of individual problem solving therapy with 8 group psycho-education sessions and a control group [11] At six months results showed that participants in both intervention arms were less likely to be depressed, both arms reported improved mental and social functioning but individual problem solving was thought to be the more effective. Using a similar group intervention to the ODIN study but comparing it only with usual care, a Norwegian study also found a group intervention to be effective in reducing depressive symptoms at 6 and 12 months [10]. Other studies showing positive results for group psychological therapy include a 12 week intervention delivered by non- medical health workers to low income Chilean women with major depression [18] and a 16 week course delivered by trained villagers in Uganda [27].

### Implications for future research

The pilot study results provide sufficient support for the funding of a larger randomised controlled trial of group based CBT in the community. The argument for a large scale trial is strong because of the increasing numbers of people with depression, the relative lack of non-medication options for GPs in the UK to help such patients, and the growing evidence that (individual) CBT is effective to treat as well as reduce relapses among such patients. Finally, similar groups are being introduced across the UK with little relevant evidence of effectiveness, and this situation needs to resolved. A systematic review of the evidence of group therapies in primary care is currently being conducted, which will complement the findings of this pilot study and help to inform a full trial.

## Conclusions

This pilot study demonstrates that a full trial of brief group CBT is feasible and that the intervention may be effective in women with depression. As an intervention offering skills training and group support, accessible to people from disadvantaged areas, the intervention has broad appeal. In an environment in which demand for mental health services is likely to continue to exceed supply, further research in this field is to be recommended.

## Competing interests

The authors declare that they have no competing interests.

## Authors' contributions

HC was involved in the design of the project, led the analysis of the qualitative data, drafted the article and revised the article. CS jointly led in the conception and design of the project, jointly assisted in the interpretation of the qualitative and quantitative data and revised the article. JC was involved in the conception and design of the project and some data interpretation. JE was involved in the design and conception of the project. RA jointly led the conception and design of the project, analysed the quantitative data and jointly assisted in the interpretation of the quantitative and qualitative data and revised the article. All authors read and approved the final version of the paper for publication.

## Pre-publication history

The pre-publication history for this paper can be accessed here:

http://www.biomedcentral.com/1471-244X/11/82/prepub
